# A DNA-PK phosphorylation site on MET regulates its signaling interface with the DNA damage response

**DOI:** 10.1038/s41388-023-02714-6

**Published:** 2023-05-15

**Authors:** Jonas P. Koch, Selina M. Roth, Aurélie Quintin, Jacopo Gavini, Eleonora Orlando, Rahel Riedo, Chiara Pozzato, Liana Hayrapetyan, Ruedi Aebersold, Deborah M. Stroka, Daniel M. Aebersold, Matúš Medo, Yitzhak Zimmer, Michaela Medová

**Affiliations:** 1 Department for BioMedical Research, Radiation Oncology, Inselspital, Bern University Hospital, and University of Bern, Bern, Switzerland; 2 Department of Radiation Oncology, Inselspital, Bern University Hospital, Freiburgstrasse 8, 3008 Bern, Switzerland; 3 Graduate School for Cellular and Biomedical Sciences, University of Bern, 3010 Bern, Switzerland; 4 Department for BioMedical Research, Visceral Surgery, Inselspital, Bern University Hospital, and University of Bern, Bern, Switzerland; 5 Department of Biology, Institute of Molecular Systems Biology, ETH Zürich, 8093 Zürich, Switzerland; 6 Faculty of Science, University of Zürich, 8057 Zürich, Switzerland

**Keywords:** Phosphorylation, Oncogenes, DNA damage response, Proteomics, Double-strand DNA breaks

## Abstract

The DNA damage response (DDR) is intertwined with signaling pathways downstream of oncogenic receptor tyrosine kinases (RTKs). To drive research into the application of targeted therapies as radiosensitizers, a better understanding of this molecular crosstalk is necessary. We present here the characterization of a previously unreported MET RTK phosphosite, Serine 1016 (S1016) that represents a potential DDR-MET interface. MET S1016 phosphorylation increases in response to irradiation and is mainly targeted by DNA-dependent protein kinase (DNA-PK). Phosphoproteomics unveils an impact of the S1016A substitution on the overall long-term cell cycle regulation following DNA damage. Accordingly, the abrogation of this phosphosite strongly perturbs the phosphorylation of proteins involved in the cell cycle and formation of the mitotic spindle, enabling cells to bypass a G2 arrest upon irradiation and leading to the entry into mitosis despite compromised genome integrity. This results in the formation of abnormal mitotic spindles and a lower proliferation rate. Altogether, the current data uncover a novel signaling mechanism through which the DDR uses a growth factor receptor system for regulating and maintaining genome stability.

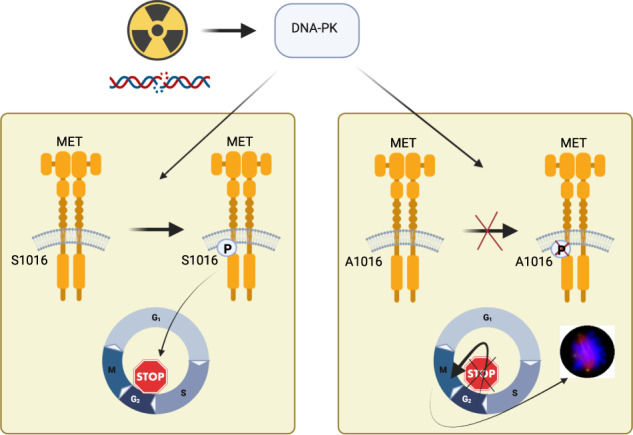

## Introduction

Radiotherapy (RT) has long been a staple of cancer therapy, but while great advances have been made in the delivery of radiation and the management of normal tissue toxicity [1], there is still a great potential to enhance its efficacy by combining it with other therapeutic means. Preclinical as well as clinical trials (reviewed in ref. [2]) have tested the combination of RT with numerous types of drugs, such as classical cytotoxic compounds, immune checkpoint modulators and oncogene-targeting therapies. Those targeted therapies aim at the inhibition of specific oncogenic signaling pathways in tumor cells and have in some cases proven useful as monotherapies to treat tumors displaying addiction to the targeted oncogene [3]. Notable pioneering examples of successful oncogene-targeting therapies include imatinib, a specific inhibitor of the chimeric BCR-ABL protein, for the treatment of chronic myelogenous leukemia, and the targeting of HER2 with the monoclonal antibody trastuzumab in breast cancer [4].

The growing body of evidence linking the deregulated activation of receptor tyrosine kinases (RTKs) to enhanced radioresistance has led to preclinical and clinical trials combining RT with RTK-targeting therapies [5]. A prominent example in this context is the case of the epidermal growth factor receptor (EGFR). In 2010, a phase III clinical trial demonstrated that EGFR inhibition by the specific monoclonal antibody cetuximab improves the benefits of RT for patients with locoregionally advanced squamous-cell carcinoma of the head and neck [6]. However, a subsequent trial showed that the addition of cetuximab to the standard chemoradiation regimen did not further improve outcome, but instead led to higher toxicity [7]. Similarly, several other trials testing various combinations of EGFR-targeting therapies, chemotherapy and RT have obtained conflicting results [5], highlighting the need for biomarkers to enable the selection of patients potentially benefiting from such combinations. A better understanding of the molecular link between RTKs and the response to irradiation could contribute to the development of stratification strategies.

Similarly to EGFR, MET is a promising candidate for targeting in combination with RT. This potent oncogene regulates cellular functions such as proliferation, survival and motility (reviewed in refs. [8] and [9]). Its deregulated expression and activation, resulting from MET receptor dimerization and tyrosine autophosphorylation on Y1234/5, have been associated with the onset and progression of several types of cancer and thus led to a great interest in the development of MET-targeting therapeutic compounds [10, 11]. Beyond the direct involvement of MET in oncogenesis via its canonical downstream signaling pathways, MET activation (either by stimulation with its ligand HGF [12, 13], or by RT [14]) has been shown to protect MET-addicted tumors from irradiation. Conversely, MET inhibition has been shown to enhance the effects of RT in vitro and in mouse xenograft models of cancer [15–17]. The specific mechanisms by which MET signaling interacts with the DNA damage response (DDR) have not been completely elucidated yet, but some insight has emerged in the recent years. Notably, several studies have shown that the pharmacological targeting of MET is associated with the formation of DSBs and impairs DNA repair, synergistically enhancing the radiosensitivity of MET-addicted tumor cells [18, 19]. Furthermore, it has been demonstrated that constitutively activated MET can regulate the nuclear translocation of RAD51, a crucial component of the DDR [20].

Recently, we have reported extensive descriptions of MET-dependent phosphoproteome in response to DNA damage [21, 22]. In the present study, we explore one unexpected DNA damage-related phosphorylation site that we extracted from this data [21]: the previously unreported Serine 1016 on MET itself (MET S1016). We show here that DNA-PK is the main kinase that phosphorylates MET S1016, unveiling a potential direct link between MET and the DDR.

We set out to explore the function of this novel MET phosphosite in the context of oncogenic MET addiction and response to irradiation. Using cellular models expressing constitutively active variants of the MET receptor, with or without a phosphodeficient mutation at the Serine 1016 position, we show that the genetic ablation of this phosphorylation affects the response to irradiation both in vitro and in vivo in a mouse xenograft model. This mutation has an impact on radiosensitivity by modulating the cell cycle response, the proliferation rate and proper mitotic spindle formation. Thus, this novel MET phosphosite stands out as the first piece of evidence for a direct connection between the DDR and MET signaling.

## Results

### DNA-PK phosphorylates MET on Serine 1016 and this phosphorylation increases upon irradiation

**Fig. 1:**
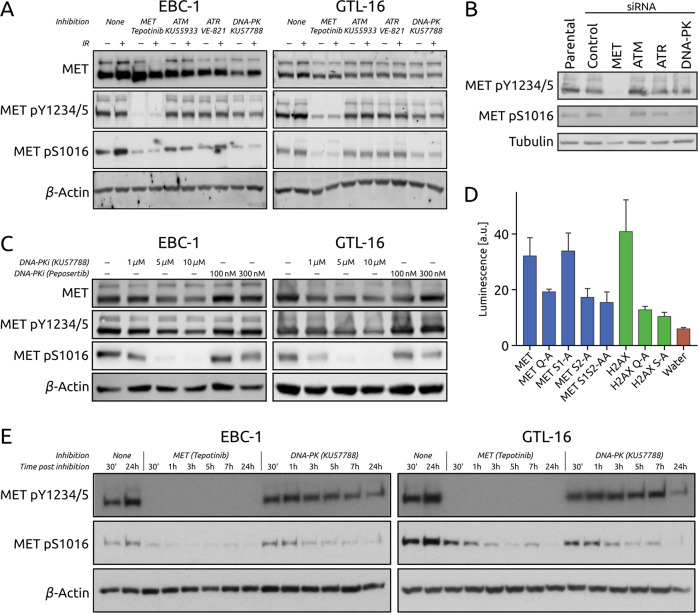
Irradiation modulates the phosphorylation of MET S1016 by DNA-PK. **A** MET total protein levels and phosphorylation levels of MET Y1234/5 and S1016 upon inhibition (24 h) of MET (tepotinib), ATM (KU55933), ATR (VE-821), or DNA-PK (KU57788) with or without irradiation (10 Gy 1 h before lysis) in EBC-1 and GTL-16 cell lines. β-Actin was used as a loading control. **B** Phosphorylation levels of MET Y1234/5 and S1016 upon siRNA-mediated silencing of MET, ATM, ATR, or DNA-PK in EBC-1 cells. Tubulin was used as a loading control. **C** MET total protein levels and phosphorylation levels of MET Y1234/5 and S1016 upon 24 h of DNA-PK inhibition by increasing concentrations of KU57788 or peposertib in EBC-1 and GTL-16 cell lines. β-Actin was used as a loading control. **D** DNA-PK in vitro kinase assay utilizing peptides corresponding to the MET S1016 region (MET: not modified; MET Q-A: mutated in the consensus sequence (SQ); MET S1-A: mutated outside of the consensus sequence, MET S2-A, MET S1S2-AA: phosphosite mutated). H2AX peptides corresponding to the S139 region (H2AX: not modified; Q-A and S-A: mutated) were used as controls. **E** MET Y1234/5 and S1016 phosphorylation levels in EBC-1 and GTL-16 cells upon MET (tepotinib) or DNA-PK (KU57788) inhibition monitored in a time-dependent manner. β-Actin was used as a loading control.

Phosphoproteomics data from MET inhibition and irradiation experiments on the MET-addicted cancer cell lines EBC-1 (lung) and GTL-16 (gastric) from [21] revealed a previously unreported phosphosite on the MET RTK: Serine 1016 (MET S1016). This serine is followed by a glutamine, which constitutes the core of the consensus sequence (SQ motif) for targets of the ATM kinase family (ATM, ATR and DNA-PK) [23], suggesting a direct intersection between MET and the DDR machinery. Using a phosphospecific antibody raised against this newly discovered phosphosite (Fig. S1), we detected this phosphorylation in untreated samples of the two *MET*-amplified cell lines EBC-1 and GTL-16 [24–26]. We also showed that the levels of MET pS1016 increased slightly upon irradiation but were strongly reduced by treatment with KU57788 (NU7441), a highly selective DNA-PK inhibitor [27] (Fig. 1A) as well as by DNA-PK siRNA treatment (Figs. 1B and S2A). At the same time, ATM and ATR inhibition by KU55933 [28] and VE-821 [29] respectively, only had a minor effect (Fig. 1A). Similarly, siRNA-mediated silencing of ATM and ATR did not reduce MET pS1016 levels (Fig. 1B). The dynamics of MET pS1016 dephosphorylation upon pharmacological inhibition of these kinases show that the phosphorylation is lost earlier when inhibiting DNA-PK than when inhibiting ATM or ATR, indicating that while ATM and ATR play a role in this phosphorylation (either directly or through their crosstalk with DNA-PK), DNA-PK seems to be the main kinase that phosphorylates S1016 of MET (Fig. S2B). We also show that MET S1016 phosphorylation decreases upon DNA-PK inhibition by KU57788 or peposertib (M3814 [30]) in a dose-dependent manner (Fig. 1C).

To confirm that DNA-PK directly phosphorylates MET S1016 we performed an in vitro kinase assay. We utilized synthetic peptides corresponding to the S1016 region with either a not modified sequence (MET), a mutation that does not disrupt the consensus SQ sequence (MET S1-A; (ASQ present instead of SSQ)), or mutations that do disrupt the SQ motif (MET Q-A, MET S2-A, and MET S1S2-AA). Analogous peptides (not modified (H2AX) and SQ motif-disrupting (H2AX Q-A, H2AX S-A)) of histone H2AX, a canonical DNA-PK substrate, were used as controls. Indeed, these data show that DNA-PK can specifically phosphorylate MET S1016 site when the consensus sequence is present (MET and MET S1-A) but not when the SQ motif is disrupted by SA (MET Q-A), AQ (MET S2-A), or AAQ (MET S1S2-AA) mutations (Fig. 1D). Interestingly, we also noticed that MET inhibition by tepotinib or by siRNA prevented both the activating autophosphorylation of MET (pY1234/5) as well as the phosphorylation of S1016 (Figs. 1A, B and S2A). Time-course experiments with MET (tepotinib) and DNA-PK (KU57788) inhibition assessing both phosphorylation sites showed that the downregulation of pS1016 is slower than that of pY1234/5 following MET inhibition whereas DNA-PK inhibition seems to downregulate pS1016 faster than pY1234/5 (Fig. 1E). Further suggesting the potential biological relevance of this phosphosite beyond MET-addicted human cancer cells, we have detected its phosphorylation upon irradiation in human cancer cell lines of various origins (Fig. S3A), and multiple sequence alignment of MET proteins from a broad selection of species showed remarkable conservation of the SQ consensus motif (Fig. S3B).

### MET Serine 1016 phosphorylation is not required for receptor autophosphorylation and recruitment of canonical MET downstream effectors

**Fig. 2:**
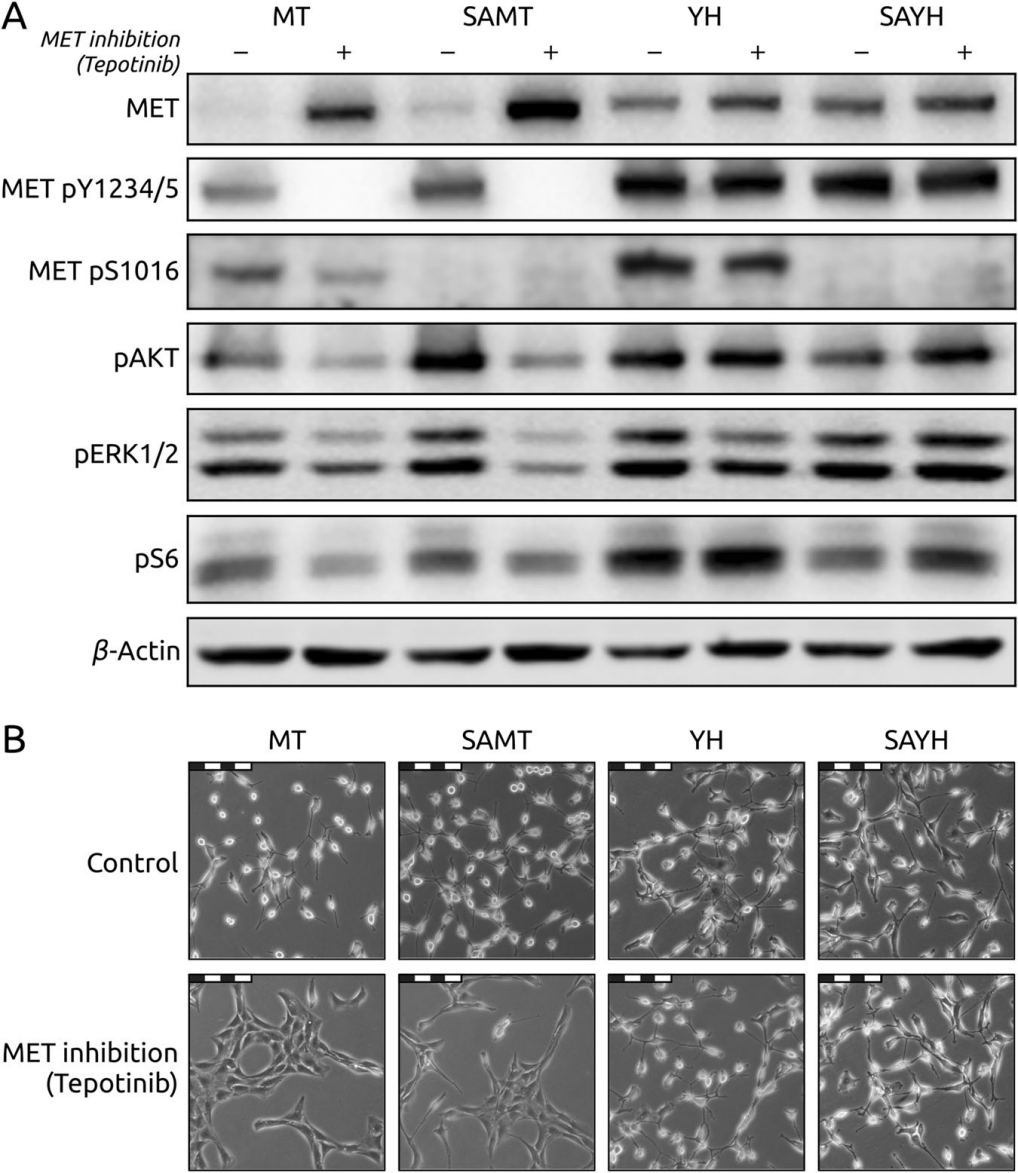
Absence of MET Ser1016 phosphorylation does not affect canonical responses to MET inhibition. **A** Western blots of whole cell lysates from NIH 3T3 mouse fibroblasts ectopically expressing murine MET with the following mutations: M1268T (MT), M1268T and S1016A (SAMT), Y1248H (YH), Y1248H and S1016A (SAYH). Antibodies for total MET, MET phosphorylated on Tyrosine 1234/1235 (active MET; MET 1234/5), MET phosphorylated on Serine 1016 (MET S1016), for downstream effectors of MET (phosphorylated forms of AKT, ERK1/2 and S6) as well as for β-Actin (loading control) were used. The SA mutation does not prevent proper activation of MET and its effectors and does not affect the sensitivity to MET inhibition (METi, 50 nM tepotinib/EMD1214063). **B** Representative pictures at 20x magnification of the cells in A (the scale bar represents 100 micrometers). The SA mutation does not prevent transformation of cells expressing active MET as seen in their more rounded, refractile morphology. The response to MET inhibition is not affected by the status of Serine 1016 either.

To assess the function of MET Serine 1016 phosphorylation, we genetically abrogated its phosphorylation in MET-dependent cellular models. We generated cell lines expressing previously described MET-mutated variants that lead to a constitutive, ligand-independent activation of the receptor and combined them with a phosphodeficient Serine 1016 to Alanine substitution. The NIH 3T3 embryonic mouse fibroblast cell line has been successfully used in the past to study the function of various MET mutations [31] and was therefore deemed as a suitable cellular system to assess the function of this newly discovered MET phosphosite. We first generated plasmids encoding two forms of constitutively active murine MET, harboring the M1268T (MT) and Y1248H (YH) mutations [31–33]. The numbering of these amino acids depends on the isoform of MET and the species. In this study, we kept the nomenclature used by Jeffers et al. to refer to these mutations [31] (see Fig. S4 for a schematic depiction of the location of these residues in human and mouse MET). Additionally, these activating mutations were combined in two additional constructs with a phosphodeficient mutation of Serine 1014 (homologous to human MET Serine 1016) to Alanine (SAMT and SAYH). For the sake of clarity, we will refer to this phosphosite as Serine 1016 for both the human and murine forms of MET. We transfected four separate pools of NIH 3T3 cells, one for each plasmid, and established cell lines from clones expressing comparable levels of the MET constructs, thus obtaining two pairs of cell lines: MT and SAMT, and YH and SAYH (Fig. S5). The introduction of the mutated constructs was confirmed by sequencing (data not shown) and the presence (MT, YH) or absence (SAMT, SAYH) of MET Serine 1016 phosphorylation by Western blotting (Fig. 2A).

While the parental cell line transfected with an empty vector expresses minimal levels of MET and no detectable active MET as assessed by the MET tyrosine phosphorylation pY1234/5 (Fig. S6A), the MT, SAMT, YH and SAYH cell lines exhibit high levels of constitutively active MET. All four cell lines also show activation (i.e. phosphorylation) of the downstream effectors of MET: AKT, ERK1/2 and S6, indicating that the phosphorylation of Serine 1016 is not required for MET autophosphorylation and recruitment and activation of its known downstream proteins (Fig. 2A). Furthermore, the status of Serine 1016 does not appear to affect the previously reported sensitivity (M1268T) and resistance (Y1248H) of the constitutively active MET variants to inhibition by ATP-competitive small molecule tyrosine kinase inhibitors (TKIs) [34]. Both the MT and SAMT cell lines respond to MET inhibition by the TKI tepotinib, as evidenced by the loss of MET tyrosine phosphorylation and the reduced phosphorylation of the downstream effectors, whereas both the YH and SAYH cell lines are resistant to MET inhibition (Fig. 2A). These observations are also confirmed on the phenotype level: all four MET-expressing cell lines display signs of cellular transformation by adopting a more epithelial, round and refractile appearance than the parental fibroblast cell line, regardless of the status of Serine 1016 (Figs. 2B and S6B). In addition, tepotinib treatment leads to the reversal to a mesenchymal phenotype solely in MT and SAMT cell lines but not in the YH and SAYH models that both harbor the MET TKI-resistant mutation YH (Fig. 2B).

### The SA mutation enhances the radiosensitivity of MET-expressing cell lines

**Fig. 3:**
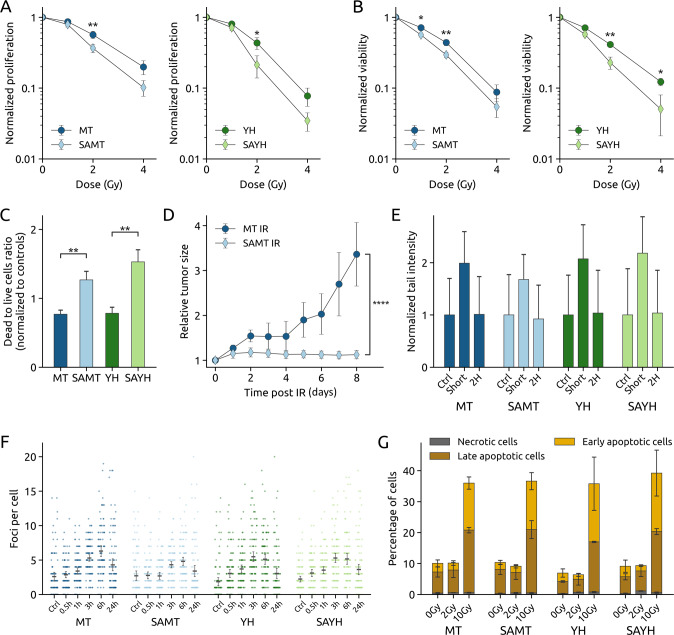
The MET S1016A mutation radiosensitizes cells in vitro and in vivo. **A**–**C** Analysis of the radiosensitivity of the MT, SAMT, YH and SAYH cell lines upon irradiation at the indicated doses with the crystal violet (proliferation), resazurin blue (viability) and Live/Dead assays, respectively. The SA mutation radiosensitizes cells expressing active MET. **D** Tumor growth (relative to size on day of treatment) of subcutaneous mouse xenografts from MT and SAMT cells upon irradiation (see Fig. S7 for a comparison with non-irradiated animals). **E** Comet assay analysis of DNA damage in the MT, SAMT, YH and SAYH cell lines, immediately (“short”) or 2 h after irradiation (10 Gy). The SA mutation has no impact on the amount of DNA damage received upon irradiation and does not prevent return to basal levels. **F** γH2AX foci analysis (100 cells per condition analyzed) of DNA damage in the MT, SAMT, YH and SAYH cell lines in untreated controls and 30 min, 1 h, 3 h, 6 h, and 24 h after irradiation by a single dose of 0,5 Gy. The SA mutation has only a slight impact on basal levels and the amount of DNA damage received upon irradiation (*p* < 0.05: MT vs. SAMT 1 h (means 3.39 vs. 2.69); *p* < 0.01: MT. vs. SAMT 3 h (5.29 vs. 4.31), 6 h (6.29 vs. 4.80); *p* < 0.001: YH/SAYH control (1.84 vs. 2.23)) and does not prevent return to basal levels. **G** Flow cytometry analysis of apoptosis induction in the MT, SAMT, YH and SAYH cell lines 4 days after irradiation at the indicated doses. Samples were analyzed for Annexin V positivity (early apoptosis) and propidium iodide (PI, necrotic cells) positivity. Double positive cells were counted as late apoptotic and negative cells as live cells. Apoptosis induction was similar for both pairs of cell lines. Statistical tests: 2-way anova (**A**, **B**), student’s *t*-test (**C**, **D**). Error bars represent the standard deviation (**C**, **D**, **F**) or SEM (**A**, **B**, **E**, **G**).

Since we have observed that DNA-PK phosphorylates MET Serine 1016 (Fig. 1) and that irradiation induces phosphorylation of this site in various MET-expressing cell lines (Figs. 1A and S3), we hypothesized that its function might be connected to the response to irradiation. We measured the radiosensitivity of the different NIH3T3-derived cell lines described above by proliferation, viability and Live/Dead assays. Interestingly, for both MET-activating mutations, the addition of the SA substitution increased the radiosensitivity (Fig. 3A–C). We further confirmed this observation in vivo by setting up a mouse xenograft model. MT and SAMT cells were implanted subcutaneously in immunodeficient mice and tumor growth was followed for 10 days after a 6 Gy single-dose treatment with local irradiation, compared to control mice. Consistent with our in vitro data, the SAMT xenografts exhibited greater sensitivity to irradiation (Figs. 3D and S7).

We next questioned how the status of MET Serine 1016 mechanistically affects the radiosensitivity. We measured the amount of DNA damage inflicted to the different cell lines by irradiation, either with a direct (comet assay) or an indirect (γH2AX foci formation) method. We could not see any significant impact of the SA substitution on the direct DNA damage infliction (Fig. 3E) or the kinetics of γH2AX foci formation and resolution apart from a trend of higher γH2AX foci counts in the MT cells in comparison to their SAMT counterparts upon irradiation (Fig. 3F). Thus, we concluded that the status of Serine 1016 does not affect the direct consequences of IR (i.e. there is not more DNA damage per Gy in the SAMT and SAYH cell lines compared to MT and YH) or the efficiency of the repair as DNA damage levels revert to basal levels quickly in all the cell lines tested. Specifically, the comet assay shows that immediately after irradiation, all cell lines display a comparable increase in DNA damage, with a prompt return to basal levels (Fig. 3E). Similarly, the γH2AX foci assay indicates a time-dependent increase in foci formation, which peaks at 3–6 h after irradiation, but no significant difference between cell lines in the resolution of foci can be observed (Fig. 3F), demonstrating the absence of difference in terms of damage detection and repair.

Therefore, we hypothesized that the radiosensitizing effect of the SA substitution might stem from an altered cellular response to DNA damage and subsequent cell death. We measured apoptosis induction after irradiation in the four cell lines by flow cytometry (Annexin V/propidium iodide staining) but could not detect a stronger induction of apoptosis in the Serine 1016-mutated cell lines. Low dose irradiation does not significantly induce apoptosis in any cell line, and high-dose irradiation causes similar levels of apoptotic cells in all of them (Fig. 3G).

### Phosphoproteomics reveals a profound impact of the SA substitution on the long-term cell cycle response to irradiation

After excluding that MET S1016 has any direct effect on DNA damage generation or repair and apoptosis, we resorted to phosphoproteomics to further explore the mechanisms underlying the radiosensitizing effect of the SA substitution. We performed a shotgun mass spectrometry analysis of the phosphoproteome of the MT, SAMT, YH, SAYH cell lines, unchallenged and after irradiation (1 h and 7 h after a single dose of 10 Gy). We specifically looked for phosphorylation changes due to irradiation that are different between the MT/YH cell lines and the SAMT/SAYH cell lines. To identify the differentially expressed phosphopeptides, we used the Python package ProtRank which has been developed especially for the analysis of proteomic and phosphoproteomic data [35].

**Fig. 4:**
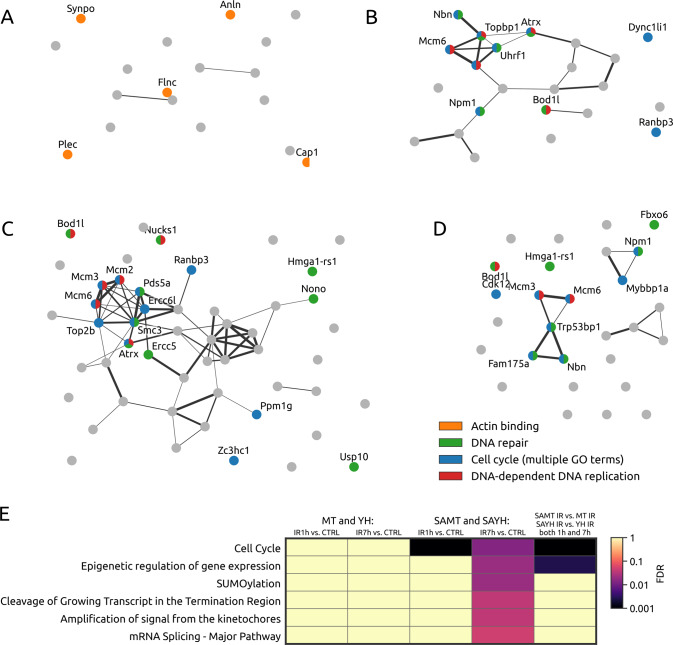
Global phosphoproteomic analysis of the MT/YH and SAMT/SAYH cells: interactions of proteins corresponding to the differentially expressed peptides and their enrichments. **A** A joint comparison of unchallenged MT vs. SAMT and YH vs. SAYH cells. **B** SAMT and SAYH response to irradiation (controls vs. irradiated samples, 1 h after 10 Gy). **C** SAMT and SAYH response to irradiation (controls vs. irradiated samples, 7 h after 10 Gy). **D** A joint comparison of MT vs. SAMT and YH vs. SAYH (both 1 h and 7 h after 10 Gy). Colors indicate the proteins’ affiliation enriched biological processes specified in the legend. We use the FDR thresholds 0.50 in **A** and 0.25 in **B**–**D**. **E** The enrichment significance of reactome pathways for various irradiation-involving comparisons. Cell cycle emerges here as the only pathway that is significantly enriched for all comparisons involving SAMT and SAYH cell lines.

A direct comparison between the unchallenged MT/YH and the SAMT/SAYH pairs revealed that the SA substitution has very little impact on the phosphoproteome in the absence of irradiation: there are only seven phosphopeptides that significantly differ (at the False Discovery Rate (FDR) threshold of 0.25 which is the standard value that was used in our analyses) between SA and non-SA cell lines. Upon increasing the FDR threshold to 0.5, 18 significant phosphopeptides are identified (Fig. 4A). Mapping the network of the 18 corresponding differentially regulated proteins reveals differences in the cytoskeleton domain (e.g., five proteins are involved in the molecular function “actin binding” which corresponds to a significant enrichment with the FDR of 0.0016).

However, considerable differences can be observed upon DNA damage in these two pairs of cell lines. MT and YH cells have a mild response 1 h after irradiation, with no identified significantly different phosphopeptides. Similarly, these two cell lines show only a slight response 7 h after irradiation with 9 significant phosphopeptides and no enrichment of relevant molecular functions or biological processes (lists of all phosphopeptides identified in the described analyses is provided in Table S1). The observations do not necessarily indicate the MT and YH cell lines do not respond to irradiation at all, but rather that their reaction is milder (and thus below the analytical threshold), or short-lived and resolved before measurement. On the other hand, the SAMT and SAYH cell lines exhibit an intense response to irradiation with substantial changes in their phosphoproteomes both 1 h and 7 h after irradiation. The response becomes stronger with time as the number of significant peptides grows from 27 to 59 between 1 h (Fig. 4B) and 7 h (Fig. 4C) post IR. When only the irradiated samples are directly compared, MT/YH vs. SAMT/SAYH, substantial differences are found again, with 31 significantly differing phosphopeptides in 28 distinct proteins regulated solely in the cell lines lacking MET Serine 1016 phosphorylation (Fig. 4D). Crucially, cell cycle emerges as the only significantly enriched Reactome Pathway in all three comparisons involving SAMT and SAYH cell lines (Fig. 4E) with the Mcm3, Mcm6, Trp53bp1, Nbn, and Fam175a proteins corresponding to the identified phosphopeptides. The accumulated evidence for the role of cell cycle in mutated cell lines prompted us to take a closer look at the role of MET Serine 1016 in this aspect of the DDR.

### The status of MET Serine 1016 impacts cell cycle re-entry after irradiation

**Fig. 5:**
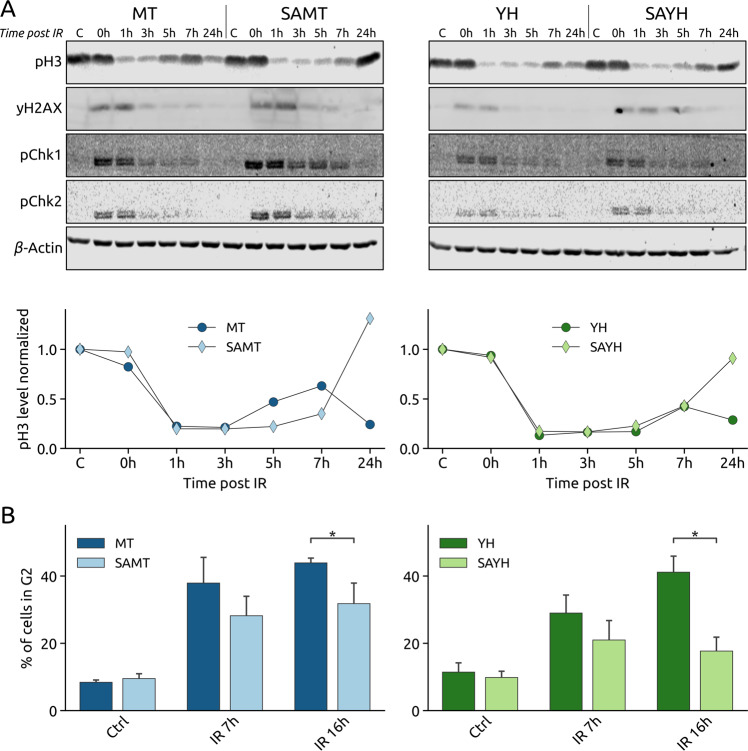
The SA mutation affects the cell cycle response to irradiation. **A** *Upper panels:* Western blots of whole cell lysates from the MT, SAMT, YH and SAYH cell lines at the indicated times (hours) after 10 Gy irradiation using antibodies for β-Actin (loading control) and the phosphorylated forms of histone 3 (H3), histone H2AX (γH2AX), Chk1 and Chk2. *Lower panels:* Quantification of the pH3 signal from the Western blots, normalized to the loading control and to the control conditions. The SA mutation leads to a slightly stronger early response to irradiation but a stronger re-entry into proliferation 24 h after treatment. **B** Flow cytometry analysis (PI staining) of the cell cycle response to IR at the indicated timepoints after 10 Gy irradiation. The SA mutation prevents proper G2 arrest after treatment (see Fig. S8 for the measurements of all phases). Statistical tests: student’s *t*-test. Error bars represent the SEM.

To assess the role of Serine 1016 with regards to cell cycle regulation, we analyzed the checkpoint response as well as the cell cycle arrest and resumption at several time points after irradiation (Fig. 5A). The early response to irradiation is similar in all four cell lines: DNA damage is rapidly detected and signaled by γH2AX, the checkpoint kinase 1 (Chk1) and checkpoint kinase 2 (Chk2) are quickly activated as inferred from their phosphorylation levels, and cells rapidly stop dividing as seen by the reduction of histone 3 phosphorylation (pH3). However, despite the appropriate activation of checkpoint kinases, a striking difference is observed 24 h after irradiation where the SA substitution seems to alter the re-entry into proliferation, as shown by the higher pH3 levels (Fig. 5A). To gain a more detailed view of the cell cycle distribution in response to irradiation, we performed a flow cytometry analysis by propidium iodide staining. We observed that the SA substitution enables cells to partially bypass the IR-induced G2 arrest, presumably resuming cell cycle earlier than the MT and YH cell lines (Figs. 5B and S8).

### MET Serine 1016 to Alanine substitution causes abnormal mitoses after irradiation

**Fig. 6:**
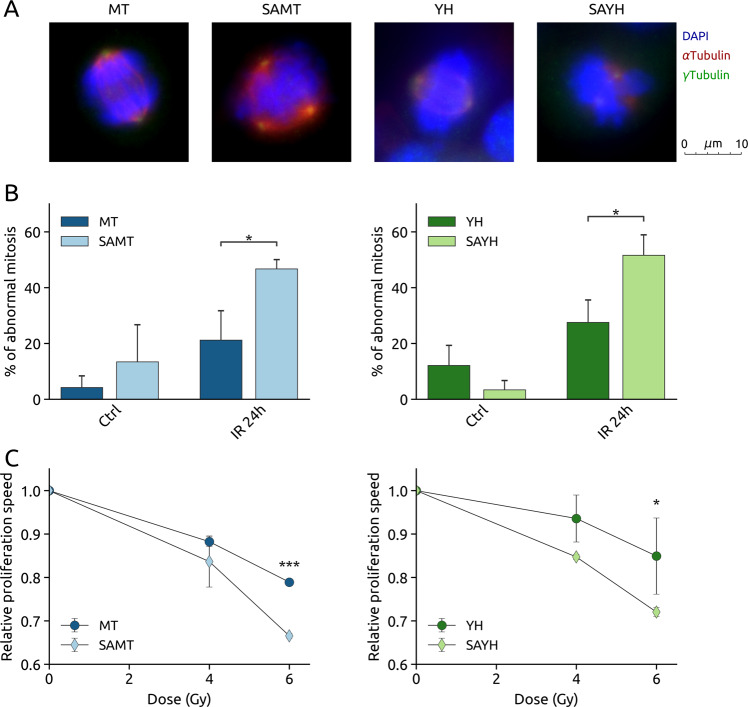
The SA mutation leads to an increase of abnormal mitoses after IR, resulting in slower proliferation. **A** Representative pictures at 60x magnification of mitoses counted as normal (MT, YH) and abnormal (SAMT, SAYH) (overlay of nuclear staining (DAPI, blue), Alpha-Tubulin (red), Gamma-Tubulin (green); single channel pictures are provided in Fig. S9). **B** Quantification of abnormal mitoses 24 h after IR (4 Gy) in the MT, SAMT, YH and SAYH cell lines. The SA mutation causes an increase in multipolar mitoses. **C** CFSE dye dilution assay for the MT, SAMT, YH and SAYH cell lines. The Relative proliferation speed was measured over 4 days after treatment with the indicated doses. The SA mutation leads to a slower proliferation after irradiation. Statistical tests: 2-way anova. Error bars represent the standard deviation (**C**) or SEM (**B**).

Proliferation measurements performed 6 days post irradiation showed that the SAMT and SAYH cells are more radiosensitive than their MT and YH counterparts (Fig. 3A). While it might seem counterintuitive that the SAMT and SAYH cells would be able to restart proliferation earlier after irradiation, we hypothesized that their greater radiosensitivity might be the consequence of a premature return to the proliferative state, leading to a lower viability at a later stage due to genomic instability. Indeed, it has been shown that improper G2 arrest after DNA damage can lead to failed mitoses resulting in tripolar spindles or chromatin bridges [36]. Therefore, it is possible that by bypassing the G2 arrest, SAMT and SAYH cells enter mitosis while their genome is still compromised, leading to mitotic failure, and explaining their lower proliferation rate. Fluorescence microscopy imaging of alpha and gamma tubulins in mitotic cells 24 h after IR revealed that, indeed, SAMT and SAYH cells display approximately twice as many abnormal mitoses, denoted by a multipolar mitotic spindle (Figs. 6A, B and S9). Besides the increased chromosomal instability resulting from mitotic errors, it has been reported that the progeny of multipolar cell divisions are inviable [37], which we hypothesize can explain the increased radiosensitivity associated with the Alanine substitution of MET Serine 1016.

Since difference in apoptosis induction can be excluded (Fig. 3G), we hypothesized that the observed variation in radiosensitivity could stem from a difference in senescence. Senescence is a state of irreversible cell cycle arrest that can be induced as an alternative to apoptosis upon irradiation. We tested whether the increase in mitotic aberrations in the SAMT and SAYH cell lines after irradiation translates to a higher induction of senescence by X-Gal staining. However, this revealed that irradiation did not lead to any significant difference in senescence in any of the cell lines, regardless of the status of Serine 1016 (Fig. S10), thus ruling out the possibility that the difference in radiosensitivity lies in this pathway.

As neither apoptosis nor senescence induction can explain the effect of the status of MET Serine 1016 on radiosensitivity, we hypothesized that the increased mitotic instability upon irradiation observed in the SAMT and SAYH cell lines could result in a reduced proliferation rate. To test this hypothesis, we made use of the CFSE dye dilution assay (Fig. S11) and calculated the proliferation rate of non-irradiated and irradiated MT, SAMT, YH and SAYH cells over 4 days. Indeed, we observed that the treated SAMT and SAYH cell lines exhibit a more pronounced reduction of their proliferative rate than MT and YH cells, explaining the observed difference in radiosensitivity (Fig. 6C). Interestingly, the dye dilution assay allows to document the effect of the SA substitution over several days, highlighting an effect that could be overlooked by more traditional end-point assays focusing on a snapshot of the situation at a specific moment. The limitations of end-point assays might explain why the live/dead assay showed a relative increase of cell death in SAMT and SAYH cells after irradiation (Fig. 3C) while measuring apoptosis and necrosis did not reveal such a difference (Fig. 3G). By analyzing the change in a population over time, the dye dilution assay shows that the SA substitution reduces the proliferative rate in an irradiation dose-dependent manner, with a 4 Gy irradiation reducing the proliferation rate by 5–10%, and 6 Gy causing a 12–13% decrease.

## Discussion

Radiotherapy is one of the most common therapeutic options for cancer management, being applied for the treatment of half of all cancer patients [38]. The advances of precision oncology and personalized medicine hold promise for more effective treatment options by using oncogene-targeted therapy to radiosensitize the tumors and thus lower the required dose of irradiation [2, 39]. However, to design optimal combinations of radiotherapy and oncogene-targeted therapy, a better understanding of the functions of the targeted oncogenes in response to irradiation is required. Encouraging efforts in these directions have been made with regards to EGFR-targeting therapies, starting with early studies indicating the radiosensitizing effects of such therapies in preclinical models [40], and supported by some clinical trials presenting the benefits of adding the anti-EGFR antibody cetuximab to RT or genotoxic chemotherapy [6, 41]. However, the inconsistent clinical results regarding such combinations are a further indication of the need to perform stratifications based on molecular data. The growing body of evidence for a direct interaction between EGFR and proteins involved in the DDR (such as DNA-PK) offers the mechanistic rationale supporting further clinical evaluation of the radiosensitizing effect of EGFR inhibition [5, 42]. Similarly to EGFR, the MET RTK is a promising candidate for targeted therapy, notably for gastric, lung, and head and neck cancer, where MET overexpression or amplification can often be observed [43]. However, the potential use of MET-targeting therapies for tumor radiosensitization has not been fully explored yet, despite the emerging evidence that MET signaling can affect the DDR in cellular and animal models [16, 44]. To evaluate the relevance of MET-targeting therapies in combination with radiotherapy, a better understanding of the MET-DDR crosstalk at the molecular level is crucial. In this context, our discovery that DNA-PK can phosphorylate MET on Serine 1016 presents a compelling direct connection between the DDR and MET signaling.

In the present study, we show that the previously unreported phosphorylation of MET Serine 1016 is induced upon irradiation in cancer cell lines of various origins. We also demonstrate that this phosphorylation is decreased following pharmacologic inhibition of DNA-PK in the gastric cancer cell line GTL-16 as well as the lung cancer cell line EBC-1, two MET receptor-addicted cancer cell lines characterized by an amplified *MET* gene copy number [24–26]. We examine how the cellular response to irradiation is affected by the abrogation of this phosphorylation, using cell lines expressing constitutively active forms of murine MET with or without a phosphodeficient Serine to Alanine mutation at this site (SAMT/SAYH and MT/YH cell lines, respectively). Our results show that preventing the phosphorylation of this serine reduces the cellular viability and growth after irradiation, both in vitro and in vivo in a mouse xenograft model (Fig. 3A–D). Contrary to our expectations, the function of MET Serine 1016 is not related to the induction of senescence or apoptosis after irradiation (Figs. S10 and 3G) and does not affect either the formation or the resolution of DNA damage (Fig. 3E, F). However, a phosphoproteomic analysis of the impact of MET Serine 1016 substitution on the response to irradiation highlighted a profound effect on the cell cycle regulation (Figs. 4 and S8). This result indicates that the relationship between MET Serine 1016 phosphorylation and the detected phenotype is complex and cannot be readily revealed by a single assay. Based on this finding, we further report that the abrogation of the MET Serine 1016 phosphorylation enables cells to re-enter the cell cycle early after irradiation and to bypass a G2 arrest (Fig. 5). This results in an increased number of mitotic errors, denoted by a multipolar spindle apparatus (Fig. 6A, B), which could lead to a higher genomic instability and explain the decreased proliferation rate (Fig. 6C). Indeed, the observation of multipolar spindles and slower proliferation coincides with reports mechanistically linking the presence of extra centrosomes with chromosomal instability, and the finding that the progeny of cells undergoing multipolar division often exhibits a loss of viability or undergoes cell cycle arrest [37].

Altogether, our data support a model in which preventing the phosphorylation of MET Serine 1016 impairs the proper cellular response to DNA damage by enabling cells to proceed through the cell cycle despite DNA damage, leading to genomic instability. Interestingly, a closer look at our phosphoproteomics data reveals differential regulation of proteins involved in spindle assembly and chromatid separation (for example Ercc6l, Smc3, Pds5a and Stmn1), hinting at a potential deregulation of the mitotic machinery. This coincides with our observation that cells lacking MET Serine 1016 enter mitosis earlier after irradiation but tend to form more numerous aberrant spindles. Such defects in the spindle assembly checkpoint and the formation of multipolar spindles are known to lead to aneuploidy [45], which can reduce cell proliferation [46]. Additionally, we can observe a lasting modulation of the phosphorylation of Mcm3 and Mcm6 after irradiation in cells lacking MET Serine 1016, suggesting a deregulation of the firing of origins of replication, yet another source of compromised genome integrity [47]. Moreover, factors involved in the stabilization of replication forks were detected in our analysis (Bod1l, Topbp1), suggesting that in the absence of MET Serine 1016, cells attempting DNA replication in the presence of residual irradiation-induced DNA damage are subject to increased replication stress, an additional factor associated with increased genomic instability [47, 48]. While our phosphoproteomic study illustrates the response to irradiation specific to cells lacking Serine 1016, whether the modulated phosphoproteins are the cause or the consequence of premature mitosis and genomic instability cannot be determined, in part because several of the detected phosphorylation sites have no assigned function yet. Thus, our study warrants further characterization of these proteins to uncover their roles downstream of MET.

Moreover, our findings pertain mainly to MET-addicted cells with high MET amplification and MET-activating mutations, yet this level of MET dependency is often not observed in cancers with lower level of MET amplification or MET overexpression. As MET dependency has considerably affected the success and failure of MET-targeted clinical trials, additional studies are required to understand the role of MET Ser1016 phosphorylation in HGF-dependent cancers or cancers with lower levels of MET amplification.

In conclusion, our results reveal that the novel DNA-PK-targeted MET phosphosite Serine 1016 is an important regulator of the cell cycle response to irradiation in cellular models featuring constitutive, ligand-independent MET receptor activation due to the presence of MET-activating mutations. Despite the apparent functional activation of checkpoint kinases, cells lacking this phosphosite seem to avoid a lasting G2 arrest and instead proceed with the cell cycle in unfavorable circumstances, leading to abnormal mitoses and a reduced proliferation rate, possibly as a result of increased genomic instability. The impact of the phosphorylation status of MET Serine 1016 in the response to irradiation is underlined by the alteration of the phosphorylation status of diverse key proteins of the cell cycle machinery, DNA repair and DNA replication. While MET inhibition could be a means to radiosensitize MET-addicted tumors, since we have shown here that genetically preventing the DNA-PK-related phosphorylation of MET Serine 1016 radiosensitizes MET-addicted cancer cells, we hypothesize that this could be a rationale to study the use of DNA-PK inhibitors to radiosensitize MET-addicted tumors (selecting patients according to the phosphorylation status of MET Serine 1016 could support a refined stratification strategy). The application of DNA-PK inhibitors to radiosensitize solid tumors poses the challenge of potentially increasing radiotoxicity for healthy tissue, but MET-addicted tumors might exhibit a stronger radiosensitization, which could favorably extend the therapeutic window of radiotherapy and other DNA-damaging agents.

## Materials and methods

### Inhibitors

If not indicated otherwise, tepotinib/EMD1214063 (Merck KGaA, Darmstadt, Germany) was dissolved in DMSO and used at a final concentration of 50 nM. KU55933 (ATM inhibitor), VE-821 (ATR inhibitor) and KU57788 (PRKDC (DNA-PK) inhibitor) (all Selleck Chemicals, Houston, Texas, USA) were used at a final concentration (f.c.) of 10 µM if not specified otherwise. DNA-PK inhibitor peposertib/M3814 (Merck KGaA, Darmstadt, Germany) was used at final concentrations of 100 nM and 300 nM. Inhibitors were dissolved in DMSO and working solutions were prepared freshly and remained in the media for the duration of the respective experiment.

### In vitro kinase assay

PRKDC (DNA-PKcs) in vitro kinase assay on synthetic peptides was performed using the ADP-Glo^TM^ system (#V4107, Promega, Madison WI, USA) along with the DNA-PKcs Kinase Enzyme System (#V4106, Promega) according to manufacturer’s instructions. The following peptides corresponding to the MET Ser1016 region were used as substrates: DQFPNSSQNG (“WT”), DQFPNSSANG (“Q-A”), DQFPNASQNG (“S1-A”) DQFPNSAQNG (“S2-A”), DQFPNAAQNG (“S1S2-AA”). As a control for the reactions, we used peptides corresponding to Ser139 region of H2AX: KKATQASQEY (“WT”), KKATQASAEY (“Q-A”), and KKATQAAQEY (“S-A”). Each reaction condition was independently set up and measured three times.

### Cell lines maintenance

Human gastric carcinoma cell line GTL-16 (provided by Dr. Paolo Comoglio (Medical School University of Torino, Italy)) and the non-small cell lung cancer cell line EBC-1 (provided by Dr. Silvia Giordano (University of Torino, Torino, Italy)) were cultured in RPMI medium (GIBCO, Invitrogen) supplemented with 5% and 10% FCS, respectively, and antibiotic-antimycotic (penicillin 100 U/mL, streptomycin sulfate 100 U/mL, amphotericin B 0.25 mg/mL; Gibco). NIH 3T3 cells (provided by Dr. Laura Schmidt (NCI, Bethesda, MD, USA)) were grown in DMEM medium (Gibco) supplemented with 10% FCS (Sigma), antibiotic-antimycotic (penicillin 100 U/mL, streptomycin sulfate 100 U/mL, amphotericin B 0.25 mg/mL; Gibco) and puromycin (Sigma, 1.5 μg/mL). Profiling of the EBC-1 cell line was done by using highly polymorphic short tandem repeat loci in June 2020 (Microsynth), the GTL-16 cells and NIH 3T3 mutants have been authenticated by whole-exome sequencing and transcriptomic analysis. All cell lines have been regularly tested for mycoplasma contamination.

### siRNA-mediated knockdown of MET, ATM, ATR, and DNA-PK

siRNAs targeting MET (Cat. # L-003156-00-0005), ATM (Cat # L-003201-00-0005), ATR (Cat. # L-003202-00-0005), DNA-PKcs (Cat #L-005030-00-0005), and the non-targeting siRNA control pool (Cat. #D-001810-10-20) were purchased from Dharmacon.

siRNAs were transfected using TransIT-X2® Dynamic Delivery System (Cat. #MIR6004) according to manufacturer’s instructions.

After seeding and adherence for 24 h, EBC-1 cells were transfected with 25 nM MET, ATM, ATR, and DNA-PK siRNAs or with 25 nM of non-targeting siRNA control. The cells were harvested for western blot analysis 48 h (non-targeting control, MET, ATM, ATR) or 96 h (DNA-PK) post siRNAs transfection.

After seeding and adherence for 24 h, GTL-16 cells were transfected with 25 nM MET siRNA or 50 nM DNA-PK siRNA and with non-targeting siRNA control, 25 nM or 50 nM, respectively. The cells were harvested for western blot analysis either 48 h (control siRNA and MET siRNA) or 72 h (control siRNA and DNA-PK siRNA) post siRNAs transfection.

### Plasmids and NIH-3T3 cells transfections

Site-directed mutagenesis was performed with the QuikChange Lightning kit (Stratagene) according to the manufacturer’s instructions. The mutations described in the main text were inserted into the pBabe puro c-met WT plasmid (gift from Joan Brugge, Addgene plasmid #17493; http://n2t.net/addgene:17493; RRID:Addgene_17493) [49]. NIH 3T3 cells were transfected with Lipofectamine™ 2000 (Invitrogen) and clones were selected with puromycin (Sigma). In all the experiments, clonal cell lines derived from a single cell each have been used. Clones expressing identical levels of total MET as well as equal basal MET Y1234/5 autophosphorylation have been employed (Fig. S5).

### Western blotting and antibodies

Protein extracts were prepared as previously described [34], separated by SDS-PAGE and blotted onto PVDF membranes. After incubation with primary antibodies (see below), signal was detected using fluorescent secondary antibodies (LI-COR). Antibodies used: pS1016 MET (CST, custom-made rabbit polyclonal, see Fig. S1 for data on antibody validation), β-Actin (Millipore, MAB1501) total MET (CST, 3127), p-Y1234/5 MET (CST, 3126), p-AKT (CST, 9271), p-ERK1/2 (CST, 4370), p-S6 (CST, 4858), pH3 (Millipore, 06-570), γH2AX (Millipore, 05-636), pChk1 (CST, 2341), pChk2 (CST, 2661). Blots were quantified with ImageStudio Lite 5.2.5. All experiments were performed independently at least three times, representative blots are shown.

### Proliferation assay

Cells were plated in triplicates in 24-well plates (300 cells/well) and treated as indicated one day after plating. Six days after treatment, cells were fixed and stained with 2% crystal violet (Sigma) in acetic acid-methanol (2:1). Cell density was measured with ImageJ. Experiments were repeated three times.

### Viability assay

Cells were plated in triplicates in 24-well plates (300 cells/well) and treated as indicated one day after plating. Six days after treatment, cells were incubated with resazurin blue (Sigma, final concentration: 3 µM) for two hours before measuring the fluorescent signal (excitation: 545 nm, emission: 590 nm). Experiments were performed in three biological replicates.

### Live/Dead assay

The assay was performed as recommended by the manufacturer (ThermoFisher). Briefly, cells were stained with Calcein AM (live cells, green) and ethidium homodimer-1 (dead cells, red) 48 h after a 2 Gy irradiation. Cells were imaged with a fluorescence microscope (Leica) and analyzed with ImageJ. Experiments were repeated independently three times.

### Mouse xenograft

20’000 cells of the indicated cell lines were injected into the flank of immunocompromised Rag2^-/-^ yc-/- mice (8-12-week-old). Tumor growth was followed daily by caliper measurement of the width, length, and depth (raw tumor sizes on each day for each experimental animal are provided in Table S2). Once tumors had reached the appropriate size, mice were randomly attributed to the control or treatment (local single-dose 6 Gy irradiation using XStrahl 150 (XStrahl Limited)) groups. Each group included 3 males and 3 females. Investigators performing treatments and tumor size measurements were blinded to group allocation. Animal experiments were approved by the local experimental animal committee of the Canton of Bern and performed according to Swiss laws for animal protection (animal license nr. BE13/13).

### Comet assay

Comet assay was performed according to the manufacturer’s instructions (Trevigen), tail intensity was measured for 50 cells/condition with the Comet assay IV program (Andor Technology). Experiments were repeated independently three times.

### Fluorescence microscopy

Cells were plated on 8-chamber microscopy slides and treated as indicated. Cells were fixed with 4% formaldehyde (Sigma), permeabilized with 0.2% Triton-X100 (Sigma) and blocked with 3% goat serum (Dako). After incubation with the appropriate primary antibody, the signal was detected with fluorescent secondary antibodies (ThermoFisher), the cells were counterstained with DAPI (Sigma, 300 nM). The coverslips were mounted with Vectashield antifade mounting medium (Vector laboratories). Imaging was performed with a fluorescence microscope (Leica). γH2AX foci formation was quantified with CellProfiler. Antibodies used: γH2AX (CST, 9027 S), α-tubulin (Sigma, T6199), γ-tubulin (Sigma, T3559). The quantification and statistical analysis of 100 cells per condition (γH2AX foci) or a triplicate of experiments (cell division) is shown.

### Apoptosis assay

Apoptosis induction was measured by flow cytometry with a FITC-Annexin V/propidium iodide kit according to the manufacturer’s instruction (Invitrogen). Acquisition was performed on an LSR II (BD Biosciences) and analysis was performed with FlowJo (FlowJo, LLC). The quantification and statistical analysis of a triplicate of experiments is presented.

### Cell cycle analysis

Cell cycle distribution was measured by flow cytometry. At the appropriate timepoint after treatment, cells were collected and fixed in 70% EtOH, washed with PBS and stained with a propidium iodide (20 μg/mL, Sigma)-RNAse A (40 μg/mL, Qiagen)-Triton-X100 (0.2%) solution in PBS. Acquisition was performed on an LSR II (BD Biosciences) and the data were analyzed with FlowJo (FlowJo, LLC). Experiments were performed three times.

### CFSE dye dilution assay

Cells were stained according to the manufacturer’s instructions (CellTrace CFSE, Invitrogen) before plating. One day after plating, cells were treated as indicated. Samples were collected daily for 5 days, starting on the day of treatment. Signal intensity was measured by flow cytometry with an LSR II (BD Biosciences) and analysis was performed with FlowJo (FlowJo, LLC). Signal intensity was plotted as a function of time, and the proliferation rate was obtained by calculating the slope of the linear regression of this function. The statistical analysis was performed on a biological triplicate of experiments.

### Discovery phosphoproteomics

Cell cultures were washed, scraped in phosphate-buffered saline, and spun down for 5 min at 1000 rpm. Resulting pellets were resuspended in 8 M urea solution containing 0.1 M ammonium bicarbonate and disrupted by sonication. Supernatants were centrifuged at 12000 rpm for 10 min and protein concentration was determined by BCA Protein Assay (Pierce). Disulfide bonds were reduced with tris(2-carboxyethyl)phosphine at a final concentration of 5 mM at 37 °C for 30 min and alkylation of free thiols was performed with 10 mM iodoacetamide at room temperature for 30 min in the dark. The solution was subsequently diluted with 0.1 M ammonium bicarbonate to a final concentration of 1.5 M urea and digestion was done overnight at 37 °C by sequencing-grade modified trypsin (Promega) at a protein-to-enzyme ratio of 50:1. Acidification was performed by adding formic acid to a final pH <3 to stop protein digestion. Peptides were desalted on a C18 Sep-Pak cartridge (Waters) and one-tenth of the resulting eluate was processed individually for total proteome analysis. Phosphopeptides were enriched from 1 mg of initial peptide mass with TiO2 as previously described [50]. For mass spectrometry analysis, samples were resuspended in 20 μl of 2% acetonitrile, 0.1% formic acid, and 1 μl of each sample was used for injections. LC-MS/MS analysis was performed with an Easy nLC 1000 system (Thermo) connected to an Orbitrap Elite mass spectrometer (Thermo) equipped with a NanoFlex electrospray source. Peptides were separated on an Acclaim PepMap RSLC C18 column (150 mm × 75 μm, 2 μm particle size, Thermo) using a gradient of 5–30% buffer B (98% acetonitrile, 2% water, 0.15% formic acid) over 180 min at a flow rate of 300 nl/min. The Orbitrap Elite was operated in data-dependent acquisition mode, each cycle consisting of one MS scan followed by 15 MS/MS scans of the most abundant precursor ions. Collision-induced dissociation was performed with the following settings: isolation width, 2 m/z; normalized collision energy, 35; activation time, 10 ms. Acquired MS data files were subsequently processed for identification and quantification using Maxquant version 1.5.2.8. Settings were kept as default with the following specifications: “First search peptide tolerance” was set to 50 ppm and “Main search peptide tolerance” to 10 ppm. Variable modifications considered were oxidation (Met) and phosphorylation (Ser/Thr/Tyr). “Label free quantification” and “Match between runs” were enabled, with a match time window of two minutes. The search was performed against the mouse UniProt FASTA dataset UP000000589. Differential expression analysis of data from the phosphoproteomics measurements was done using the Python package ProtRank [35]. The input data comprised raw counts of 7572 phosphopeptides that have been measured in 4 samples (4 cell lines, 3 conditions: control, 1 h and 7 h after 10 Gy; two replicates for each sample). Of all raw counts, 44% were missing values (zeros). The thresholding for significantly differentially expressed phosphopeptides was done using the false detection rate (FDR) of 0.25 (unless specified otherwise). The subsequent enrichment analysis of the obtained phosphopeptides was performed using STRING (https://string-db.org/) [51]. The results are visualized as network maps: every protein is a “node”, and the predicted associations between proteins are represented by “edges” (lines of varying thickness according to the confidence of the predicted interaction). Relevant enrichments are highlighted in colors as indicated in the figure legend.

### Senescence β-galactosidase assay

Cells were plated in 6-well plates and treated as indicated. Seven days after treatment, cells were stained with X-Gal as previously described [52] and imaged with an inverted microscope. Representative pictures from three independent experiments are presented.

### Irradiation

Cells were irradiated using a Cesium^137^ source at a dose rate of 0.86 Gy/min (Gammacell 40, MDS Nordion). For the experiments assessing γH2AX foci formation, cells were irradiated by X-Rad225XL (Precision X-Ray) at a dose rate of 116.2cG/min and employing the 0,3 mm Cupper filter.

### Multiple sequence alignment

The alignment was performed on uniprot.org with the default clustalo settings: The default transition matrix is Gonnet, gap opening penalty is 6 bits, gap extension is 1 bit. Clustal-Omega uses the HHalign algorithm and its default settings as its core alignment engine. The algorithm is described in ref. [53].

### Statistical analysis

Statistical analysis was performed using Prism 7.03 (GraphPad), *p*-values < 0.05 were considered significant (*p < 0.05; **p < 0.01; ***p < 0.001). If not stated otherwise, all the experiments were performed at least three times.

## Supplementary information

Supplementary figures legends

Figure S1

Figure S2

Figure S3

Figure S4

Figure S5

Figure S6

Figure S7

Figure S8

Figure S9

Figure S10

Figure S11

Table S1

Table S2

## Data Availability

Materials described in the manuscript, including all relevant raw data, will be freely available to any researcher wishing to use them for non-commercial purposes, without breaching participant confidentiality.
