# Supplementary material for: A DNA-PK phosphorylation site on MET regulates its signaling interface with the DNA damage response
Source: Oncogene. 2023 May 15;42(26):2113–25. doi: 10.1038/s41388-023-02714-6 (PMC10289896; doi:10.1038/s41388-023-02714-6)
Supplement: Supplementary file 1 — Supplementary figures legends [file 41388_2023_2714_MOESM1_ESM.docx]

**Supplementary figures legends**

**Supplementary Figure 1: Validation of the anti-phospho S1016 MET antibody**. Western blots of whole cell lysates from GTL-16 cells treated with MET inhibitor for 24h (METi), 10Gy irradiation 1h before lysis (IR), or both (Combo). Membranes were probed with the affinity purified anti-pS1016 MET serum (1st panel), the serum with a non-phosphorylated blocking peptide (corresponding to the region around MET S1016) to remove signal from non-phosphorylated MET (2nd panel), the serum with the same peptide as the 2nd panel but phosphorylated on S1016 to block all signal (3rd panel), a total MET antibody to confirm the size of the signal detected (4th panel). The serum alone detects the same signal as a total MET antibody, saturating the serum with a non-phosphorylated blocking peptide only detects MET when phosphorylated on S1016, and the signal is stronger upon irradiation, as expected for a putative ATM/ATR/DNA-PK target. The serum saturated with the non-phosphorylated blocking peptide is referred to as “pS1016 MET antibody” throughout this paper for the sake of simplicity and was made fresh for every blot.

Supplementary Figure 2: Modulation of MET Serine 1016 phosphorylation upon inhibition of MET and DNA damage response master kinases. (A) MET S1016 phosphorylation levels in GTL-16 cells upon siRNA-mediated knockdowns of MET (*left* *panel*) and DNA-PK (*right*). Controls were transfected with a non-targeting siRNA pool and tubulin was used as a loading control. (B) MET S1016 and Y1234/5 phosphorylation levels in EBC-1 and GTL-16 cells upon ATM, ATR or DNA-PK inhibition monitored in a time-dependent manner (1, 7 and 24 hrs of inhibition). β-Actin was used as a loading control.

Supplementary Figure 3: MET Serine 1016 is phosphorylated upon irradiation in various MET-expressing cell lines and is conserved across species. (A) Western blots of whole cell lysates from MET-expressing cancer cell lines of various origins (Hs746t: gastric adenocarcinoma, UMSCC-10B: laryngeal squamous cell carcinoma, HN5 and SCC-61: Tongue squamous cell carcinoma, A-431: epidermoid carcinoma) using antibodies specific for β-Actin (loading control) and for human MET phosphorylated on Serine 1016. MET Serine 1016 phosphorylation was induced in all cell lines 1 hour after irradiation with 10 Gy (IR +) compared to low basal phosphorylation in untreated cells (IR -). (B) Multiple sequence alignment of the 120 amino acid peptide sequence around MET Serine 1016 of various species. The alignment was performed with the Clustal-Omega program with default parameters: the default transition matrix is Gonnet, gap opening penalty is 6 bits, gap extension is 1 bit. Clustal-Omega uses the HHalign algorithm and its default settings as its core alignment engine.

**Supplementary Figure 4: Schematic map of MET phosphosites and mutation sites.** This drawing shows the respective location of relevant amino acids for human MET (canonical and non-canonical isoforms) and murine MET. The UniProt identifiers are displayed in brackets. Downregulating phosphorylation sites are shown in red, activating phosphorylation sites in green. Sites mutated in our cell lines with constitutive active MET (MT and YH mutations) are shown in blue, and Serine 1016 in orange.

**Supplementary Figure 5**: **Comparison of basal total MET as well as MET pY1234/5 levels in the MT, SAMT, YH and SAYH cell lines.** Western blots of whole cell lysates from MT, SAMT, YH and SAYH cell lines, untreated (-) or 1h after 10 Gy irradiation (+). β-Actin was used as a loading control.

**Supplementary Figure 6:** **MET-related signaling and morphology in control and tepotinib-treated NIH 3T3 cells expressing the empty vector construct.** **(A)** Western blots of whole cell lysates from NIH 3T3 mouse fibroblasts ectopically expressing empty vector construct used to express murine MET-mutated variants. Levels of total MET, MET phosphorylated on Tyrosine 1234/1235 (active MET; MET 1234/5), MET phosphorylated on Serine 1016 (MET S1016), and MET downstream effectors (phosphorylated forms of Akt, ERK1/2 and S6) with and without MET inhibition (METi; 50 nM tepotinib/EMD1214063) are shown. β-Actin was used as a loading control. **(B)** Representative pictures at 20x magnification of the cells in A (the scale bar represents 100 micrometers). Fibroblast-like morphology of the NIH 3T3 cells expressing the empty vector does not change by MET inhibition treatment.

Supplementary Figure 7: MT and SAMT xenografts growth. Growth curves of MT and SAMT tumors with and without IR treatment (growth curves corresponding to the irradiated groups of animals are reproduced from Figure 3D). Statistical tests: 2-way anova. Error bars represent the standard deviation. Raw data of tumor sizes are reported in Supplementary Table 1.

Supplementary Figure 8: Cell cycle distribution of MT, SAMT, YH and SAYH cells after a 10 Gy single-dose irradiation. Error bars represent the standard deviation.

Supplementary Figure 9: Mitoses in MT, SAMT, YH and SAYH cells. Representative pictures at 60x magnification of mitoses counted as normal (MT, YH) and abnormal (SAMT, SAYH). Single-channel pictures of nuclear staining (DAPI, blue), Alpha-Tubulin (red) and Gamma-Tubulin (green).

Supplementary Figure 10: Status of Serine 1016 does not affect senescence induction upon irradiation. Representative pictures at 10x magnification (the scale bar represents 200 micrometers) of β-Gal activity in the MT, SAMT, YH and SAYH cell lines 7 days after irradiation with the indicated doses. The read arrowheads show positive cells. The SA mutation does not influence the induction of senescence, which remains low in all cell lines after treatment.

Supplementary Figure 11: Schematic representation of the CFSE dye dilution assay.

Before plating, the cells are stained with CFSE, a fluorescent compound that covalently binds the proteins inside the cells. During mitosis, the fluorescently tagged proteins are distributed equally between the two daughter cells, therefore reducing the strength of the signal by half for every generation. Cells are collected daily for 5 days starting on the day of treatment. The signal intensity (log2) is plotted as a function of time, and the slope of this function is the dye dilution rate. The dilution rate of treated cells is normalized to their respective controls to obtain the normalized proliferation rate of different cell populations as a function of the treatment dose.

**Supplementary Table 1: Phosphoproteomics data – lists of phosphopeptides identified in the differential analyses.**

**Supplementary Table 2:** **Raw MT and SAMT tumor sizes (mm^3^) 0 – 8 days post IR treatment.**
