# Supplementary figures and images for: A DNA-PK phosphorylation site on MET regulates its signaling interface with the DNA damage response
Source: Oncogene. 2023 May 15;42(26):2113–25. doi: 10.1038/s41388-023-02714-6 (PMC10289896; doi:10.1038/s41388-023-02714-6)

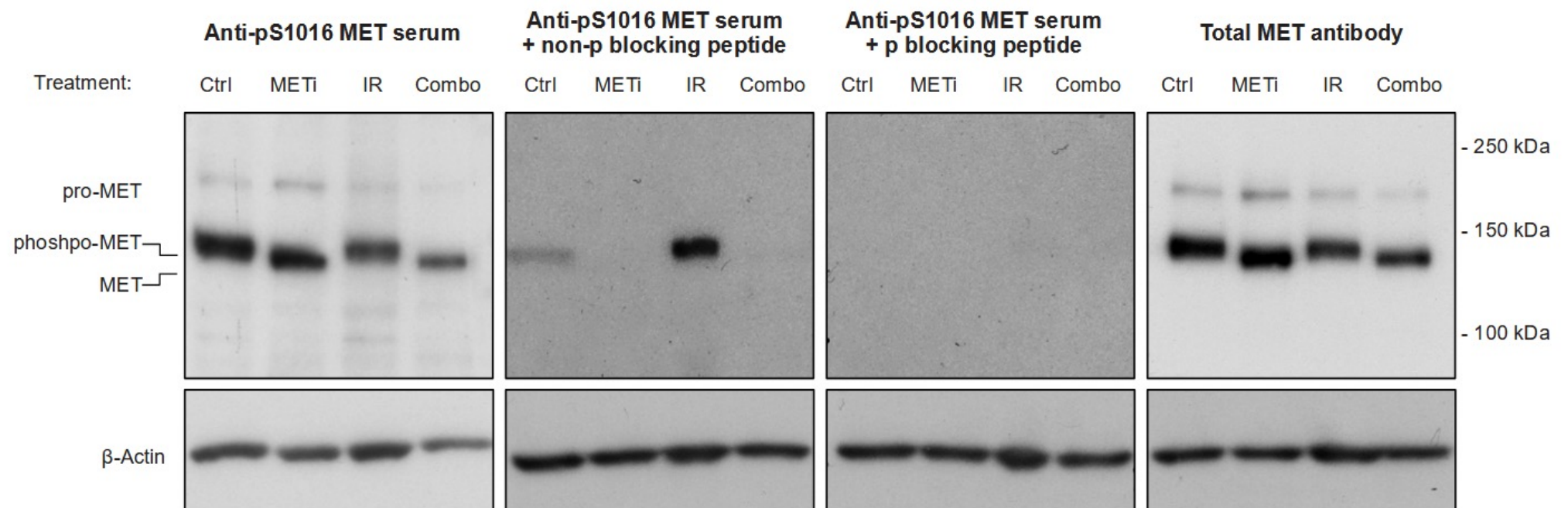

Figure S1

Supplement: Supplementary file 2 — Figure S1 [file 41388_2023_2714_MOESM2_ESM.pdf]

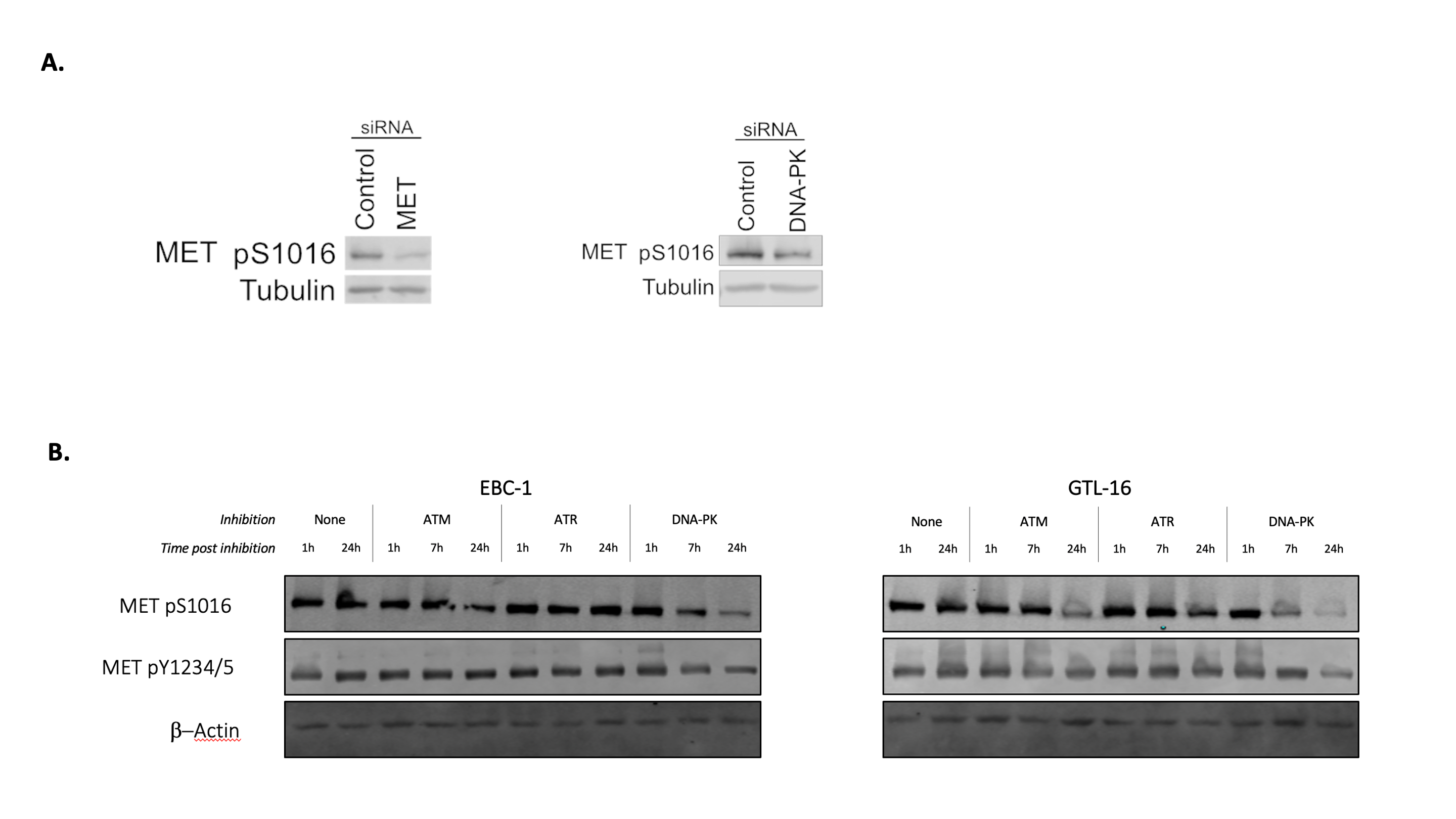

Supplement: Supplementary file 3 — Figure S2 [file 41388_2023_2714_MOESM3_ESM.png]

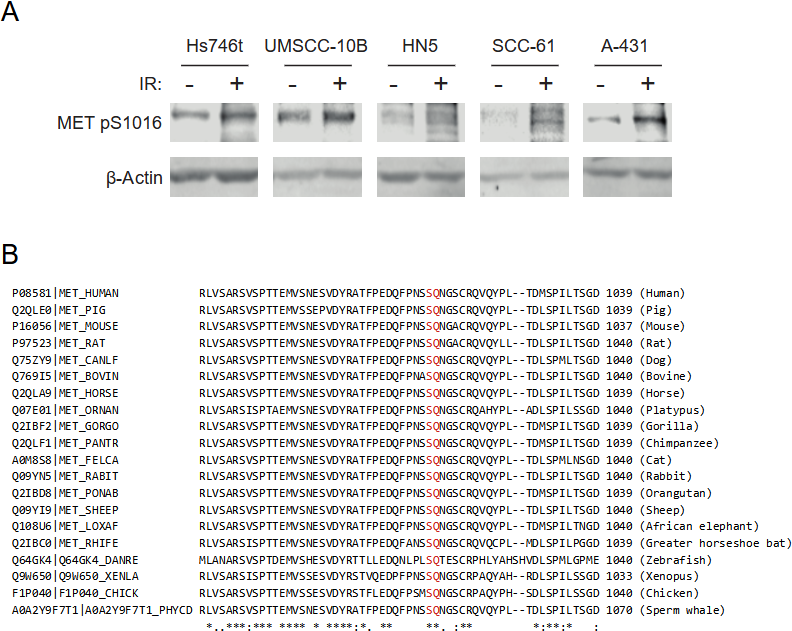

Supplement: Supplementary file 4 — Figure S3 [file 41388_2023_2714_MOESM4_ESM.tif]

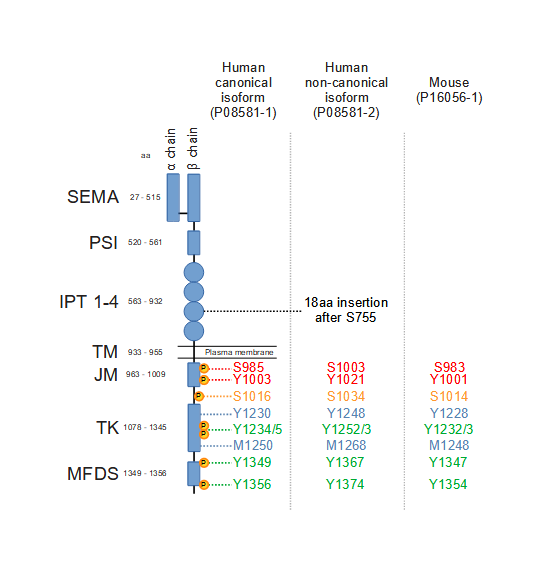

Supplement: Supplementary file 5 — Figure S4 [file 41388_2023_2714_MOESM5_ESM.tif]

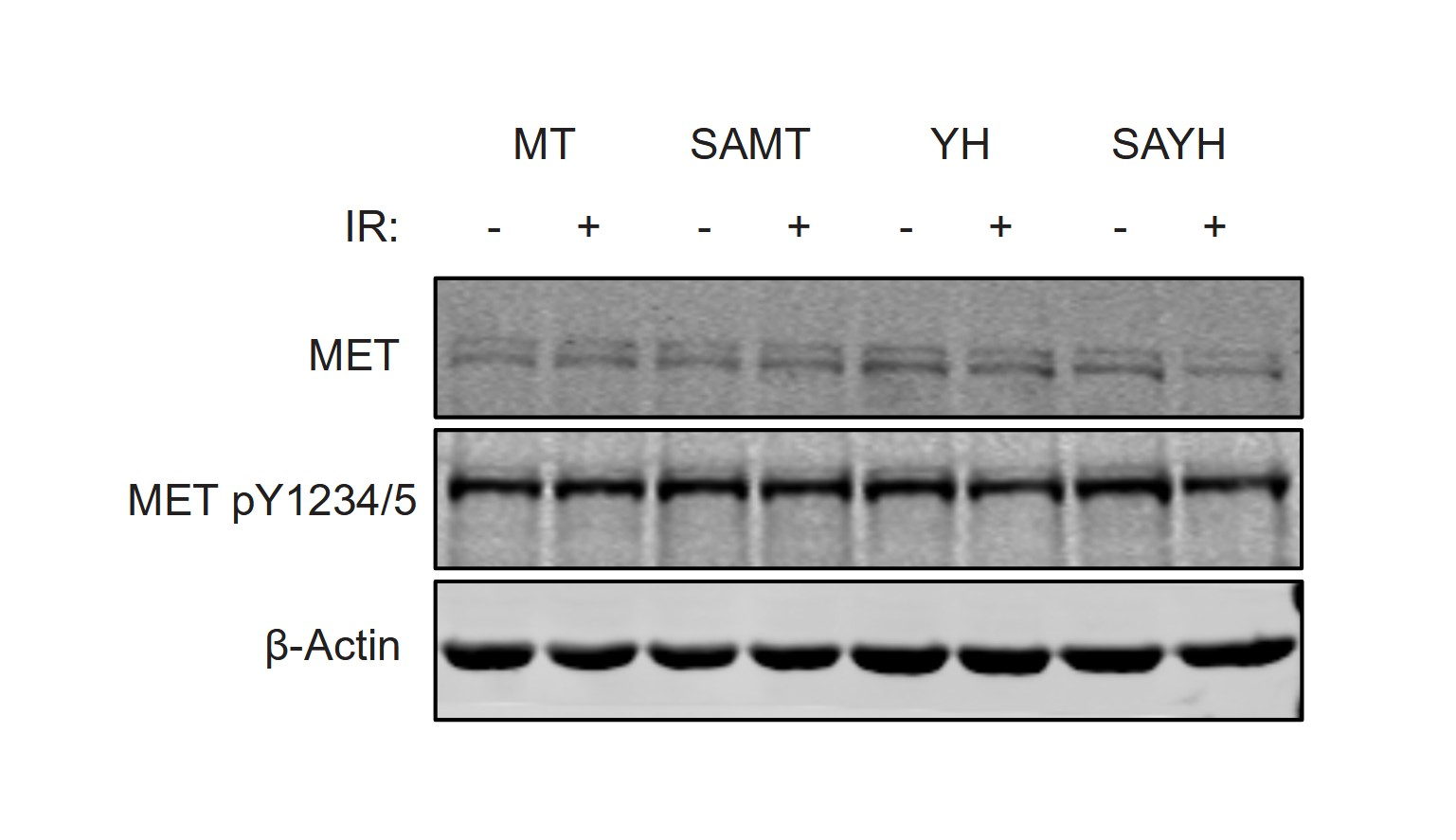

Supplement: Supplementary file 6 — Figure S5 [file 41388_2023_2714_MOESM6_ESM.tif]

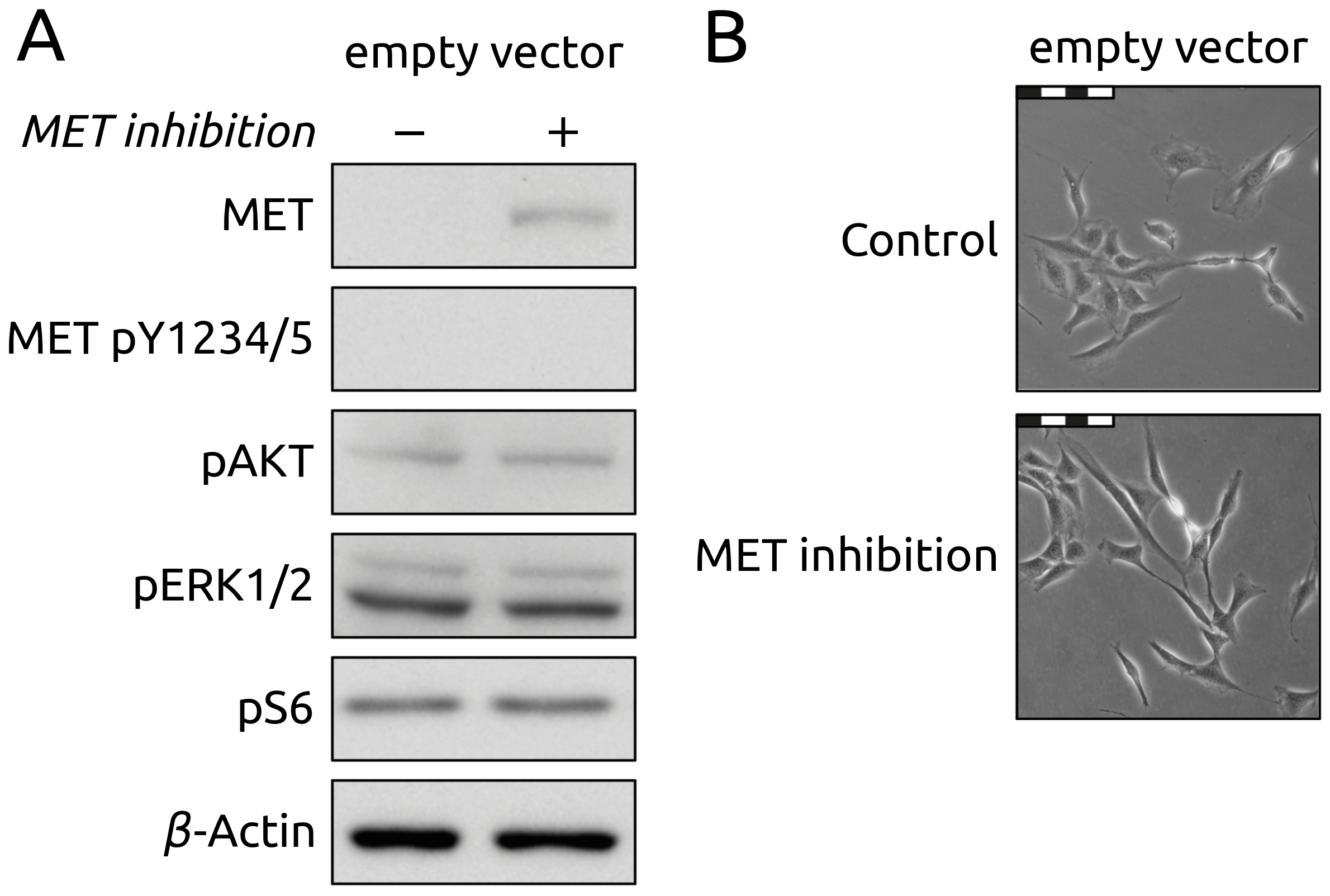

Supplement: Supplementary file 7 — Figure S6 [file 41388_2023_2714_MOESM7_ESM.png]

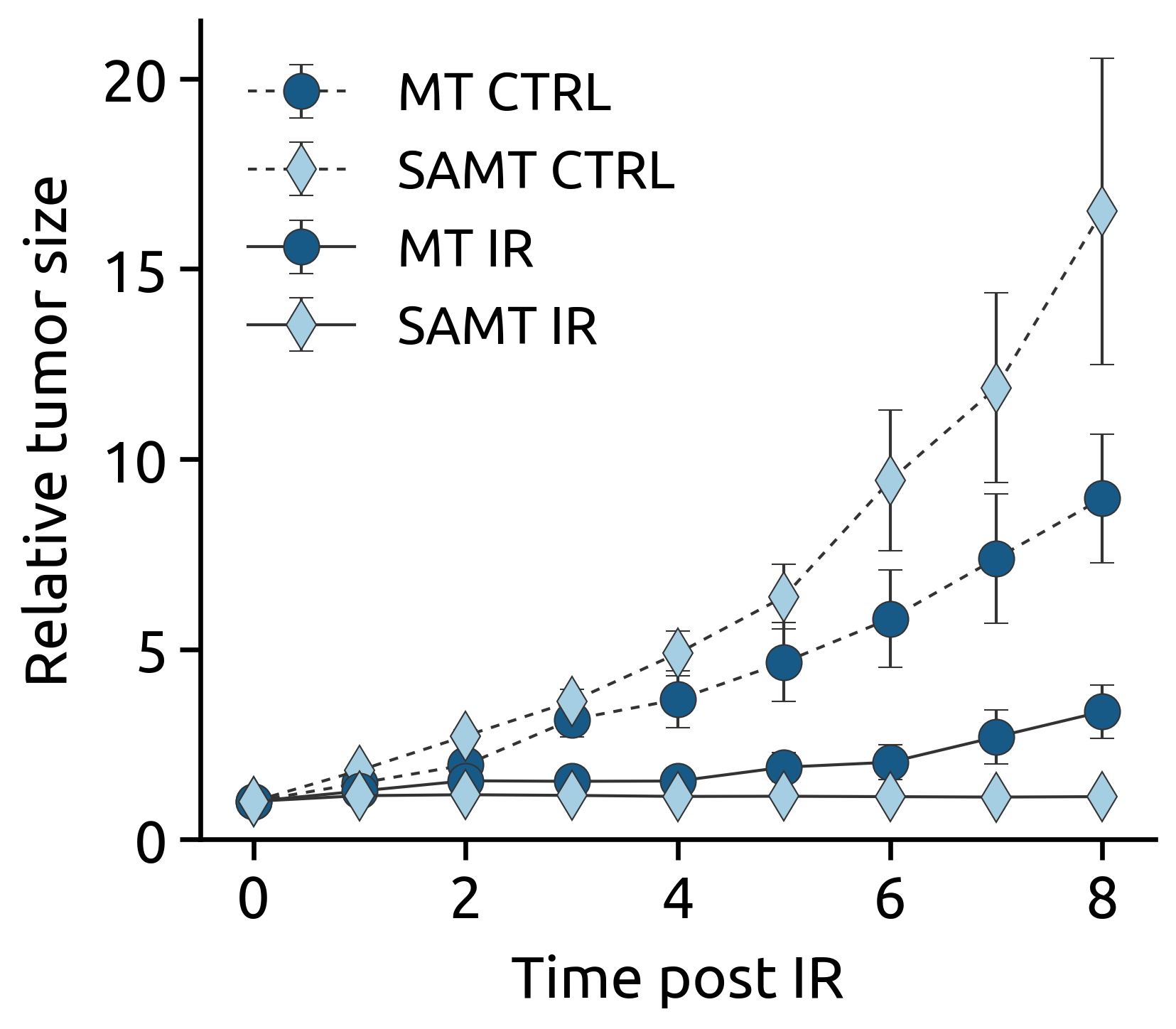

Supplement: Supplementary file 8 — Figure S7 [file 41388_2023_2714_MOESM8_ESM.png]

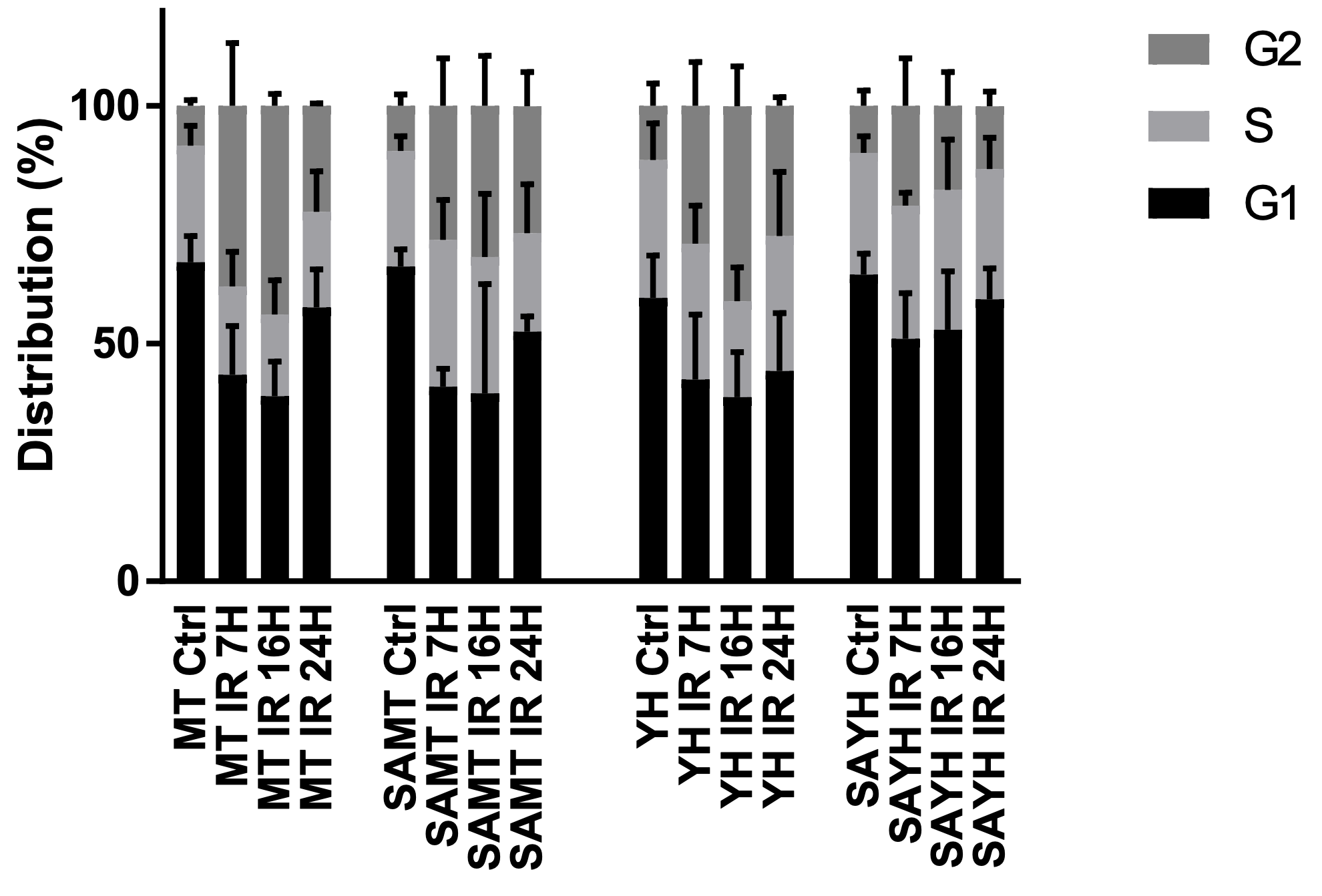

Supplement: Supplementary file 9 — Figure S8 [file 41388_2023_2714_MOESM9_ESM.tif]

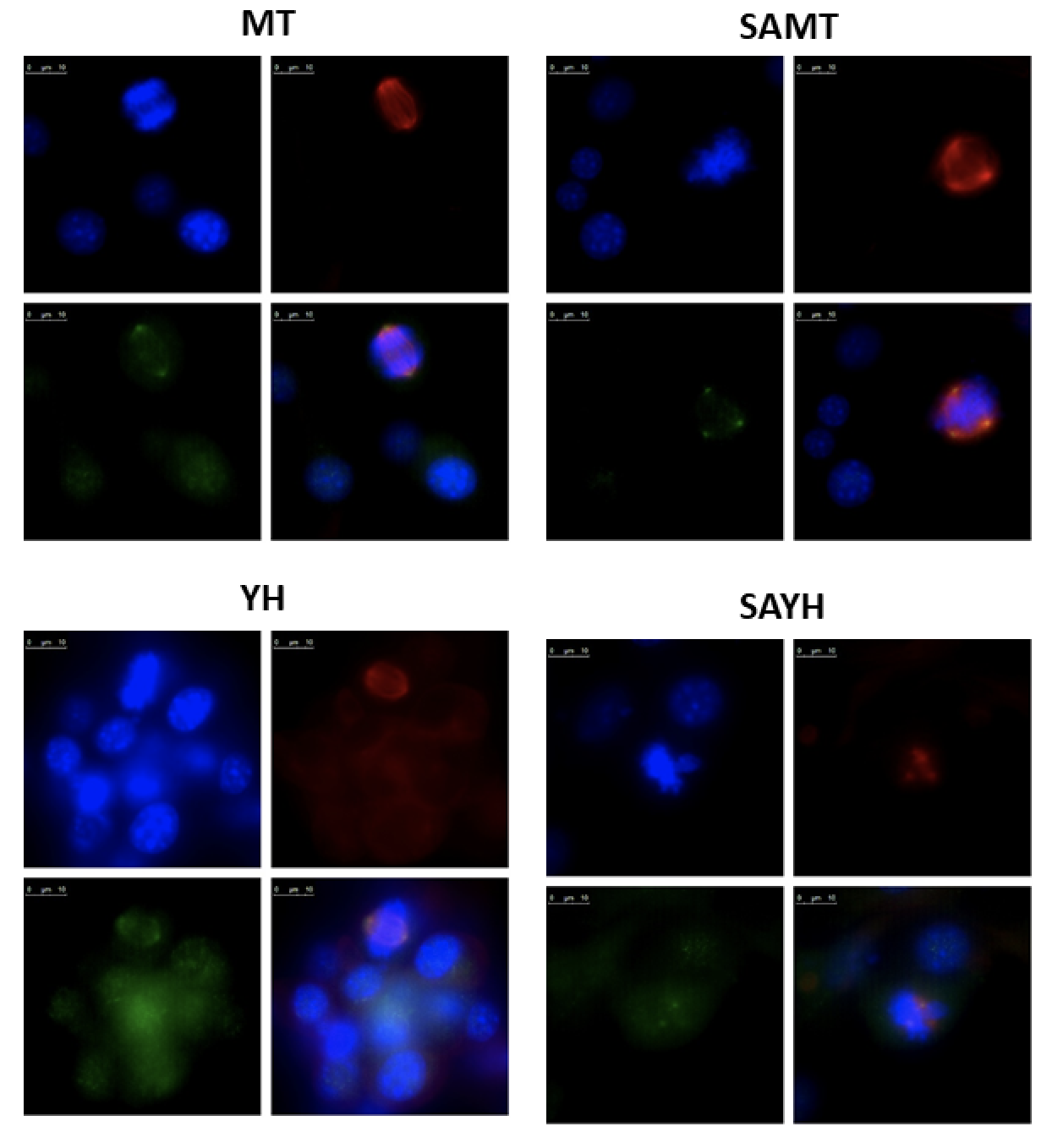

Supplement: Supplementary file 10 — Figure S9 [file 41388_2023_2714_MOESM10_ESM.tif]

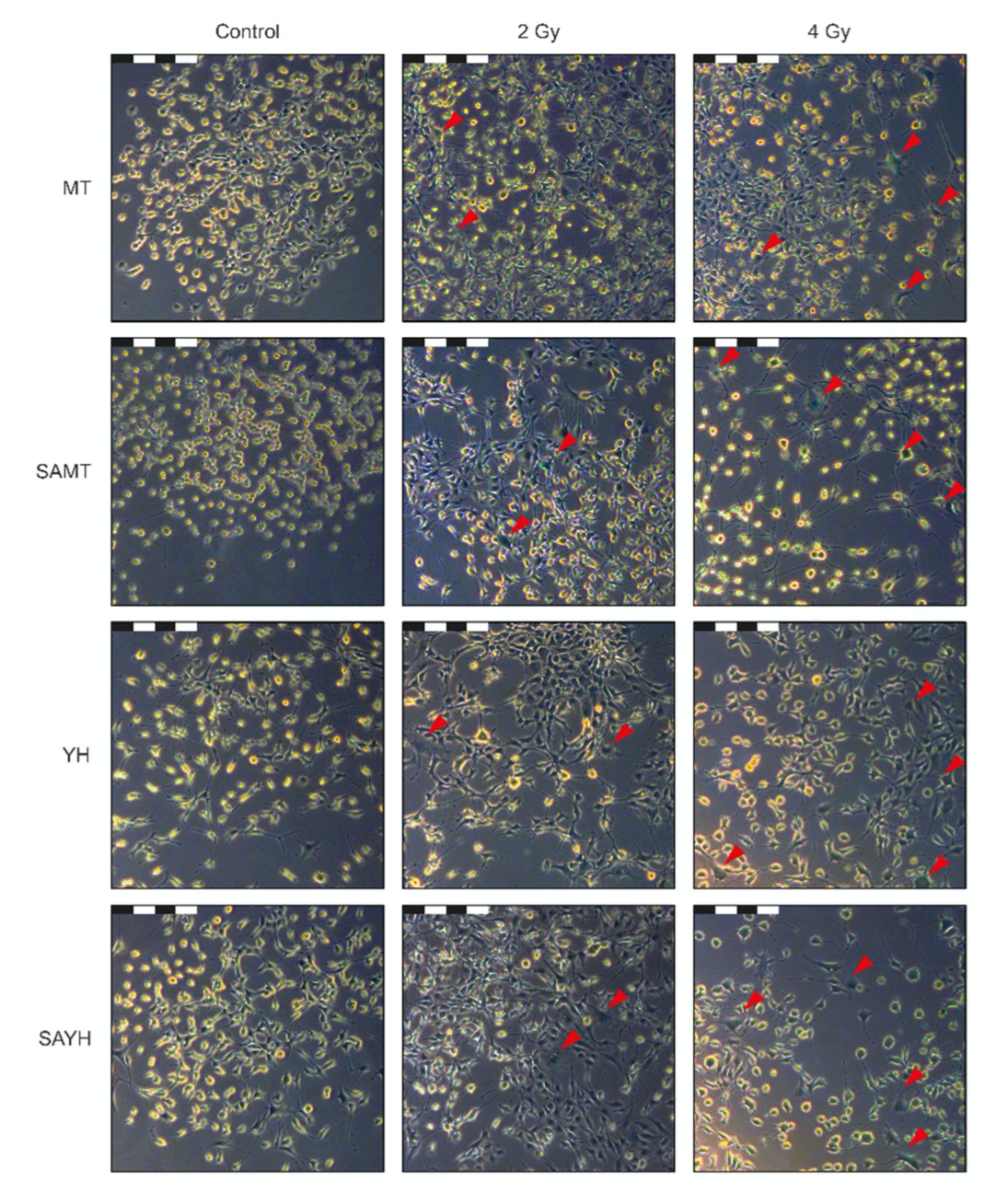

Supplement: Supplementary file 11 — Figure S10 [file 41388_2023_2714_MOESM11_ESM.tif]

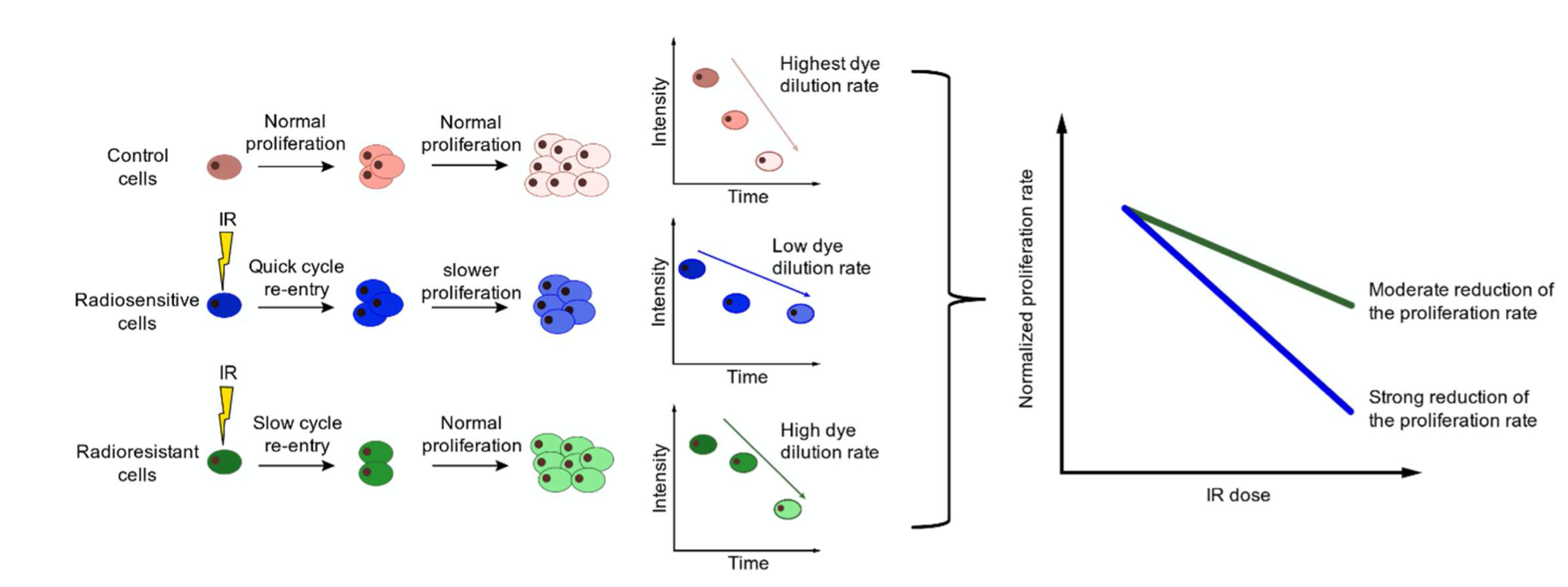

Supplement: Supplementary file 12 — Figure S11 [file 41388_2023_2714_MOESM12_ESM.tif]
